# Intracellular Aβ42 Aggregation Leads to Cellular Thermogenesis

**DOI:** 10.1021/jacs.2c03599

**Published:** 2022-05-26

**Authors:** Chyi Wei Chung, Amberley D. Stephens, Tasuku Konno, Edward Ward, Edward Avezov, Clemens F. Kaminski, Ali A. Hassanali, Gabriele S. Kaminski Schierle

**Affiliations:** † Department of Chemical Engineering and Biotechnology, University of Cambridge, Philippa Fawcett Drive, Cambridge CB3 0AS, U.K.; ‡ UK Dementia Research Institute, Department of Clinical Neuroscience, University of Cambridge, Cambridge CB2 0AH, U.K.; § Condensed Matter and Statistical Physics, International Centre for Theoretical Physics, Strada Costiera 11, Trieste 34151, Italy

## Abstract

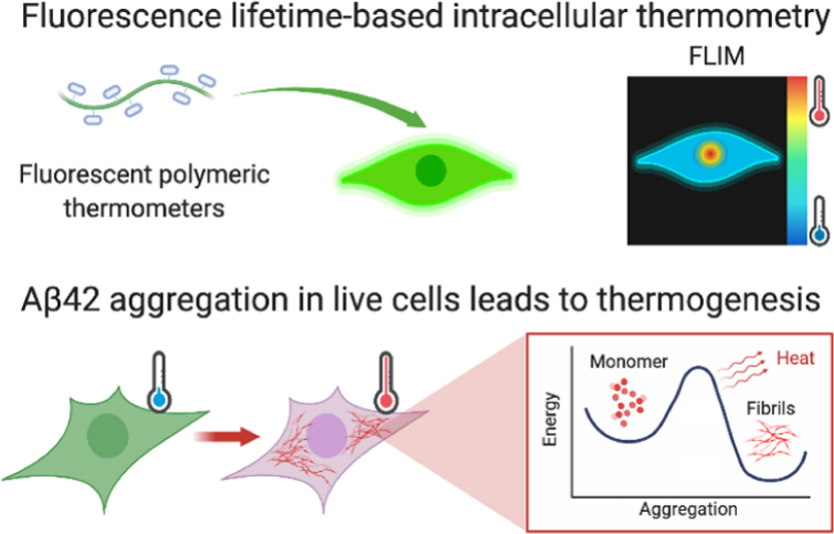

The aggregation of Aβ42 is a hallmark of Alzheimer’s disease. It is still not known what the biochemical changes are inside a cell which will eventually lead to Aβ42 aggregation. Thermogenesis has been associated with cellular stress, the latter of which may promote aggregation. We perform intracellular thermometry measurements using fluorescent polymeric thermometers to show that Aβ42 aggregation in live cells leads to an increase in cell-averaged temperatures. This rise in temperature is mitigated upon treatment with an aggregation inhibitor of Aβ42 and is independent of mitochondrial damage that can otherwise lead to thermogenesis. With this, we present a diagnostic assay which could be used to screen small-molecule inhibitors to amyloid proteins in physiologically relevant settings. To interpret our experimental observations and motivate the development of future models, we perform classical molecular dynamics of model Aβ peptides to examine the factors that hinder thermal dissipation. We observe that this is controlled by the presence of ions in its surrounding environment, the morphology of the amyloid peptides, and the extent of its hydrogen-bonding interactions with water. We show that aggregation and heat retention by Aβ peptides are favored under intracellular-mimicking ionic conditions, which could potentially promote thermogenesis. The latter will, in turn, trigger further nucleation events that accelerate disease progression.

## Introduction

Neurodegenerative diseases are a result of protein misfolding and aggregation, with aging as its prime risk factor.^1^ This includes Alzheimer’s disease (AD), where the misfolding of amyloid-β (Aβ, in particular its 42-residue variant, Aβ42) into insoluble plaques is a characteristic of the disease. The most basic model for amyloid growth is a nucleation-elongation model, with nucleation being associated with a high energy barrier.^2^ There is a prevailing hypothesis of localized hotspots, that is, energy-intensive areas, which may arise as the cell ages or during cellular stress. In these hotspots, the free energy activation barrier may be more likely to be overcome, hence spurring the initial nucleation, which subsequently facilitates monomeric addition (i.e., the elongation process). It has been shown, using in vitro isothermal titration calorimetric measurements, that Aβ42 elongation is an exothermic process^3,4^ which could lead to temperature gradients inside a cell. Mitochondrial thermogenesis is a heat-releasing effect linked to mitochondrial damage, the latter being associated with cell aging and disease. Interestingly, mitochondrial thermogenesis has further been implicated in the aggregation of heat-sensitive mitochondrial proteins.^5^ Hence, it could act as another potential source of localized hotspots. It has long been established that Aβ42 interacts with, and can even localize and accumulate in mitochondria,^6,7^ thus resulting in mitochondrial dysfunction and neurotoxicity. Carbonyl cyanide *p*-(tri-fluromethoxy)phenyl-hydrazone (FCCP) is a proton uncoupler of oxidative phosphorylation which is commonly used to induce mitochondrial thermogenesis in vitro.^8,9^ It interferes the proton gradient across the electron transport chain (ETC), thereby disrupting adenosine triphosphate (ATP) synthesis. A recent study by Lautenschläger et al.^10^ has found that the addition of FCCP leads to greater intracellular Aβ42 aggregation in a HEK293T cell model. As mitochondria are the power source of the cell, it is unsurprising that oxidative stress incurred in the presence of Aβ42 aggregation could lead to changes in its metabolism, and hence to the overall energetics of the cell. Therefore, measuring intracellular temperatures is fundamental to a better understanding of the biochemical processes that may occur within a cell undergoing stress. Here, we perform intracellular thermometry using fluorescent polymeric thermometers (FPTs)^11,12^ and mitochondrial metabolism assays to investigate the intertwined pathways of Aβ42 pathology and mitochondrial dysfunction in a live cell model. The FPTs contain a fluorescence unit (*N*-{2-[(7-*N*,*N*-dimethylaminosulfonyl)-2,1,3-benzoxadiazol-4-yl](methyl)amino}ethyl-*N*-methylacrylamide; DBD-AA) which acts as a temperature sensor, as changes in its fluorescence lifetime are directly correlated to the changes in temperature. Fluorescence lifetime is more robust than fluorescence intensity readouts against experimental variability, that is, fluorophore concentration and laser intensity, and has been used for intracellular thermometry measurements previously.^13,14^

We show here that the presence of Aβ42 in live HEK293T cells leads to an average temperature increase, in addition to inhibitions of glycolytic and oxidative respiratory pathways; however, the observed thermogenesis stems from exothermic Aβ42 elongation, instead of associated mitochondrial damage. This rise in temperature can therefore be mitigated upon treatment with a newly screened small molecule drug which binds to the C-terminus of Aβ, thereby causing steric hindrance and preventing its aggregation.^15^ Lastly, using classical molecular dynamics simulations on Aβ, we show that heat dissipation from a protein is more greatly hindered in an intracellular-mimicking ionic environment compared to an aqueous one, due to altered protein–water hydrogen bonding interactions as result of ionic interactions with termini groups on the protein. Furthermore, the three-dimensional packing of the Aβ, in terms of the parallel and anti-parallel β-sheets, also affects thermal relaxation behavior of the protein.

## Results

### Intracellular Thermometry Can Detect Protein Aggregation in Live Cells

**Figure 1:**
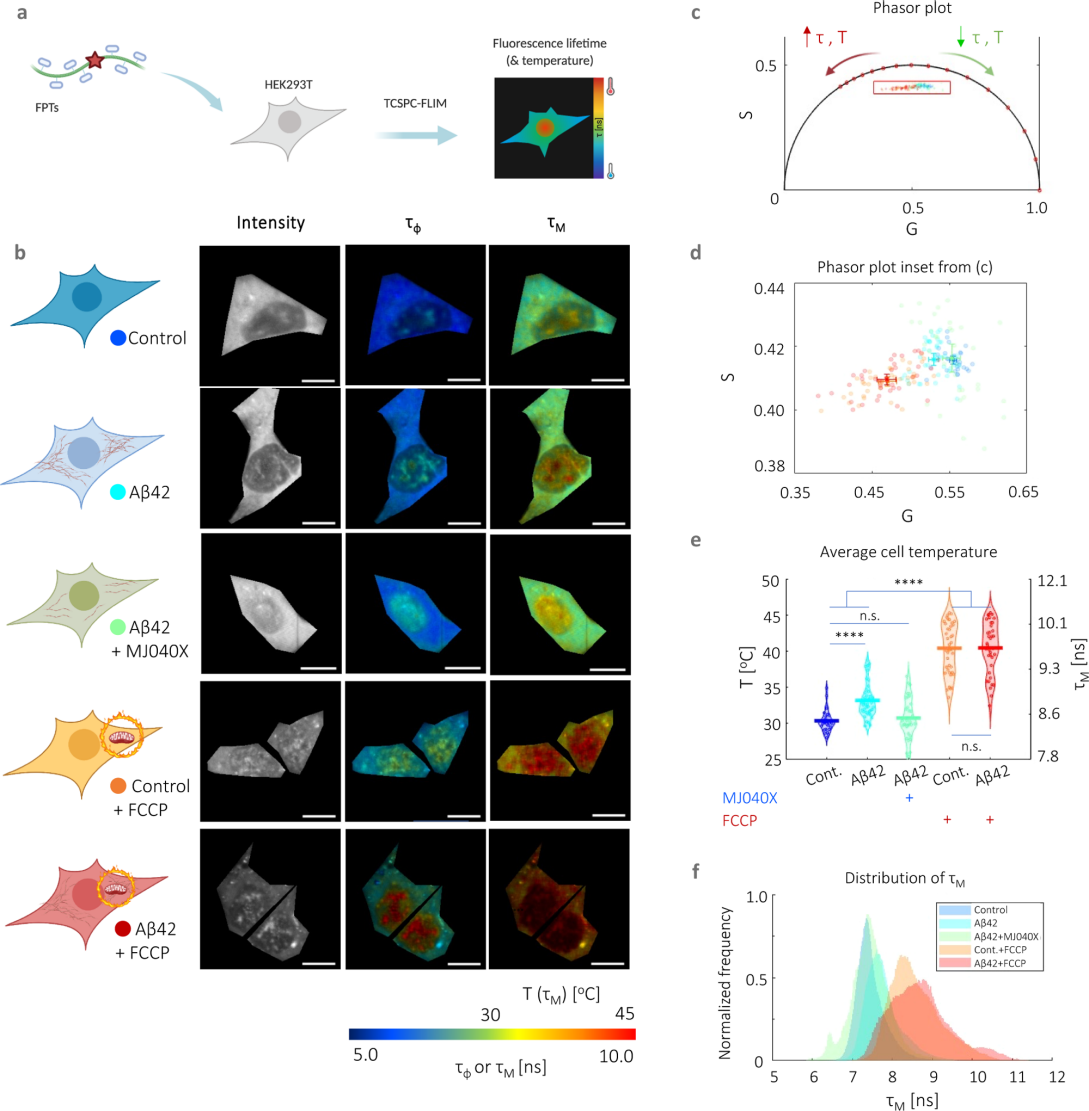
Intracellular thermometry using fluorescence lifetime-based readings from FPTs can be used as a platform for detecting Αβ42 aggregation and testing anti-aggregation drugs. (a) Cartoon representation of FPT–FLIM, where FPTs are introduced into live HEK293T for intracellular thermometry measurements based on fluorescence lifetime readouts. (b) Intracellular thermometry maps reveal the existence of intracellular temperature gradients. For each cell sample tested, their cartoon representation is shown alongside FPT–FLIM fluorescence intensity and phase (τ_φ_) and modulation fluorescence lifetime (τ_M_) images, the latter being the temperature-calibrated parameter. Scale bar, 10 μm. (c) Bi-exponential decay of FPTs is apparent from significantly different τ_φ_ and τ_M_, as well as their phasors that fall within the universal semicircle, each dot and shaded circle represent the mean phasor from a single cell and standard deviation within the sample, respectively, in the zoomed in plot (d). A legend is provided in the cartoon representations in (b). (e) Cell-averaged temperature values are given with mean and SEM average values of 30.4 ± 0.5 (control), 33.2 ± 0.8 (Aβ42), 30.8 ± 0.9 (control + MJ040X), 40.4 ± 1.2 (control + FCCP), and 40.4 ± 1.2 °C (Aβ42 + MJ040X). The presence of Aβ42 and/or FCCP elevated mean cell temperature slightly and significantly, respectively. The addition of MJ040X, which reduces the aggregation extent of Aβ42, reverts temperatures to that of the control. Thermometry experiments are conducted on >30 cells imaged over three biological repeats (i.e., *N* = 3). (f) Distribution of τ_M_. One-way ANOVA tests (with Holm–Sidak’s multiple comparisons) were performed, where n.s. is not significant, ** is *p* < 0.005, *** is *p* < 0.001, and **** is *p* < 0.0001. Cartoon representations are created on BioRender.com.

In a first set of experiments, we incubated HEK293T cells with or without 500 nM unlabeled recombinant Αβ42^16^ for 24 h prior to intracellular thermometry measurements. As additional controls, we treated cells with MJ040X, Aβ42+MJ040X, FCCP, and Aβ42+FCCP. MJ040X is the esterified version (i.e., to facilitate cellular uptake) of a recently discovered small molecule drug which sterically hinders the aggregation process by binding to the C terminus of Aβ42.^15^ Using time-correlated single photon counting (TCSPC) fluorescence lifetime imaging microscopy (FLIM) to measure temperature-dependent changes in FPTs, we observe that the presence of Aβ42 elevates average intracellular temperatures by 2.8 ± 0.6 °C (Figure 1). Using super-resolution direct optical stochastic reconstruction microscopy (dSTORM), we confirm that exogenous addition of Aβ42 monomers and overnight incubation leads to the formation of fibrillar structures with an average length of 162 nm, dispersed throughout the cytoplasm of cells (Figure S1). Furthermore, this thermogenesis effect is alleviated upon treatment with MJ040X to cells with exogenously added Aβ42, where temperature differences become insignificant from control cells which do not contain Aβ42. To confirm that MJ040X reduces the extent of Aβ42 aggregation in our current model, we used a fluorescence lifetime-based aggregation sensor, as we previously did to study amyloid aggregation in live cells.^17^ We added 10% HiLyte488 (HL488) labeled and 90% unlabeled Aβ42 to the cells prior to measuring the level of Aβ42 aggregation in the presence or absence of MJ040X. Upon the addition of MJ040X, cells with added labeled Aβ42 have a higher fluorescence lifetime of ∼0.1 ns in comparison to cells with Aβ42 without MJ040X, which indicates that Aβ42 is at a less aggregated state^17^ in the presence of MJ040X (Figure S2). Moreover, a non-cell based, in vitro assay to measure Aβ42 aggregation kinetics shows that MJ040 (i.e., the non-ester version of MJ040X and therefore the active MJ040 molecule present in the cytoplasm as the ester gets cleaved once the drug has reached the intracellular space^15^) works by inhibiting the exothermic elongation process rather than the nucleation of Aβ42 (Figure S3).

For intracellular thermometry measurements, we validated our imaging setup using Rhodamine B, a dye with fluorescence emission properties that are strongly affected by temperature^18,19^ (Figure S4a–c). We assembled a temperature-controlled setup consisting of a stage top incubator and an objective warmer to control environmental parameters precisely. We then performed a temperature to fluorescence lifetime calibration of the FPTs in live cells (Figure S4d—f). We analyzed FPT–FLIM data using phasor plot, an a priori and global approach more suited to complex exponential decays (i.e., which the FPTs possess) and low photon count images.^20,21^ In comparison to conventional exponential fitting, which relies on nonlinear least squares fitting, this involves the Fourier transform of time-domain TCSPC data into the frequency-domain, resulting in “phasors” plotted on a polar plot. The bi-exponential decay of the FPTs^11^ is apparent from the difference in phase and modulation lifetimes (Figure 1b–d), and the latter is used as a calibration parameter to temperature due to its higher sensitivity to temperature (Figure S4e). Cells with higher fluorescence lifetimes, hence higher temperatures, have phasors that move along the phasor plot in an anticlockwise manner (Figure 1c–e). The highest temperature differences (i.e., 10 ± 1.0 °C) are measured in cells with added FCCP, a protonophore, showing the severe mitochondrial thermogenesis effect due to disruption in the ETC, as expected. There is no difference between FCCP-treated cells with and without Aβ42, indicating that exothermic effects from Aβ42 aggregation is outweighed by severe dysfunction of the mitochondria as triggered by the proton uncoupler (Figure 1e,f). Thus, FPT–FLIM can capture thermal events associated with Aβ42 aggregation in live cells.

### Exothermic Elongation is the Primary Contributor to Thermogenesis in Cells with Aβ42 Aggregation

**Figure 2:**
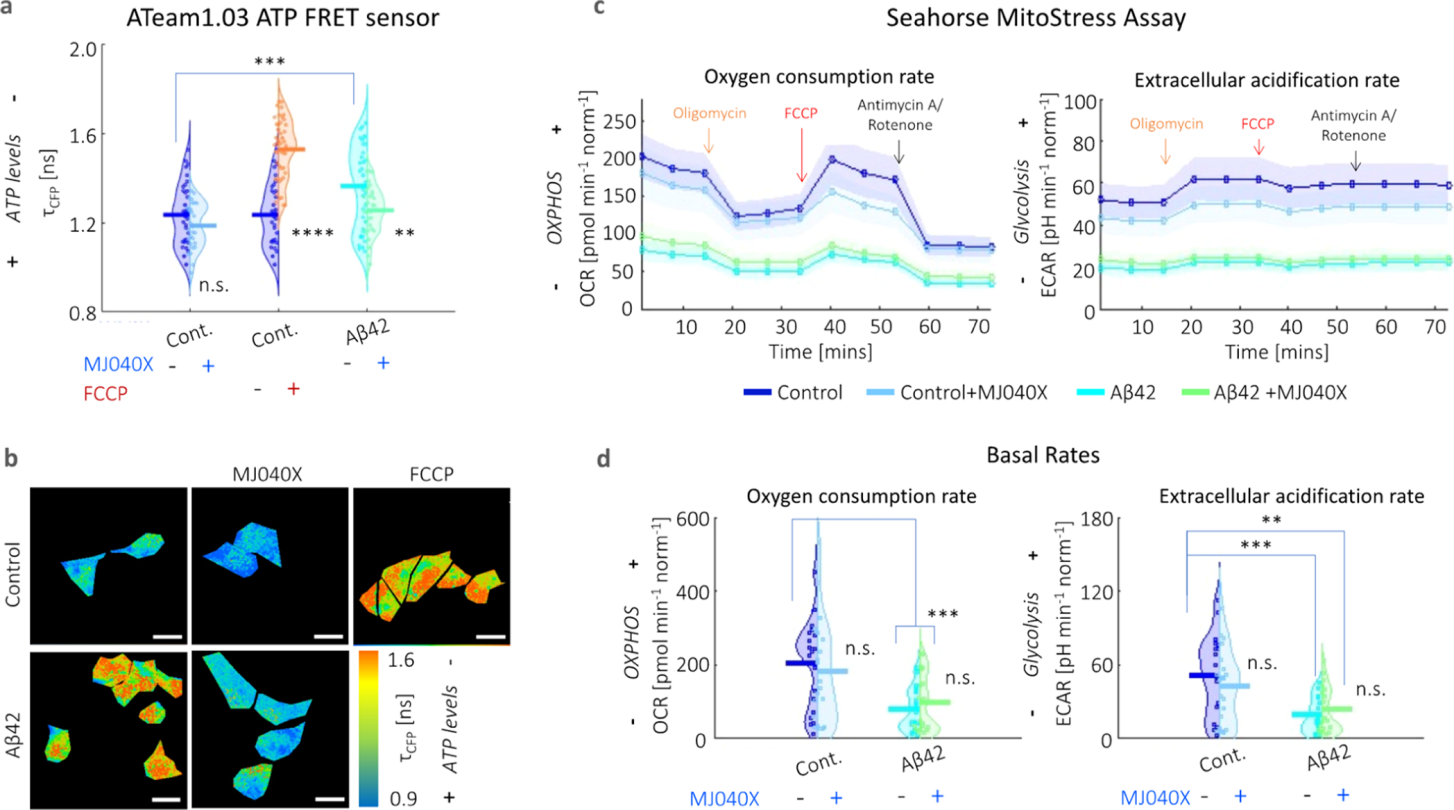
Presence of Aβ42 adversely impacts mitochondrial health. (a) Averaged fluorescence lifetimes and (b) corresponding maps of the fluorescence lifetime of the CFP donor (τ_CFP_) of the ATeam1.03 ATP sensor. Fluorescence emission decay profiles are given in Figure S5. ATP synthesis is negatively affected by the presence of Aβ42 and FCCP. The addition of MJ040X to cells with Aβ42 results in an increase in ATP production seen by the increase in τ_CFP_._._ (c) Seahorse assay shows lower OCR and ECAR (indicative of oxidative phosphorylation and glycolysis, respectively) over time in the presence of Aβ42 (cyan for Aβ42 only and green for Aβ42 + MJ040X), which prevails over the effect of MJ040X (light blue for control + MJ040X and green for Aβ42 + MJ040X) on mitochondrial metabolism. Arrows indicate when modulators are injected into cell media; this includes: oligomycin (orange), FCCP (red), and antimycin A/rotenone (black). (d) Basal OCR and ECAR [i.e., at the starting time point in (c)] are impacted by the presence of Aβ42 after 24 h of incubation. Basal OCR and ECAR are 203.7 ± 29.7, 181.8 ± 35.7, 78.7 ± 14.2, and 79.1 ± 19.4 pmol min^–1^ norm^–1^ and 51.8 ± 0.8.0, 43.1 ± 7.5, 19.4 ± 3.5, and 23.6 ± 4.7 pH min^–1^ norm^–1^, respectively, for control, control+MJ040X, Aβ42, and Aβ42 + MJ040X. ATeam1.03 analysis is based on >30 cells per sample over three biological repeats (*N* = 3), and the Seahorse assay is based on the quantification of four wells per sample over three biological repeats (*N* = 3) and are normalized by cell count. One-way ANOVA tests (with Holm–Sidak’s multiple comparisons) were performed, where n.s. is not significant, ** is *p* < 0.005, *** is *p* < 0.001, and **** is *p* < 0.0001.

By comparing the temperature elevation in the case of exogenously added Aβ42 cells, but with and without MJ040X, heating contributions can be attributed not only to exothermic elongation, as indicated above, but also to mitochondrial dysfunction. Hence, to decouple these factors, we investigate mitochondrial metabolism using two different assays. The first one involves Ateam1.03, a CFP-mVenus Förster resonance energy transfer (FRET) pair, which acts as a cytosolic ATP sensor.^22^ The presence of ATP causes more interactions between the FRET pair, which can be detected by a decrease in the donor fluorescence lifetime. CFP has bi-exponential fluorescence emission decay characteristics, which is clearly seen from its nonlinear profiles in Figure S5. The results show that Aβ42 aggregation causes a significant loss of ATP which can be rescued by the addition of MJ040X; however, in the case of the control only cell, MJ040X does not significantly affect ATP levels. In the case of FCCP addition to control cells (i.e., the positive control), there is a significant reduction in ATP synthesis, with a corresponding rise in fluorescence lifetime of ∼0.4 ns (Figure 2a,b). These results are further corroborated by a Seahorse Mito Stress assay, the gold standard technique which distinguishes between energy metabolism from contributing glycolytic and oxidative phosphorylation pathways, by the measurements of extracellular acidification rates (ECAR) and oxygen consumption rates (OCR), respectively (Figure 2c,d). When measured over time, cells with exogenously added Aβ42 with (cyan line) and without MJ040X treatment (green line) have lowered ECAR and OCR, in comparison to the control cells with and without Aβ42 (dark and light blue lines, respectively) upon the addition of modulators (e.g., oligomycin, FCCP and antimycin A/rotenone) which target different complexes forming part of the ETC (Figure 2c).

Moreover, it is interesting to note that upon exposure to Aβ42 for 24 h, the basal (i.e., initial) respiratory rates are already significantly impacted at twofold the rates of control cells without Aβ42, that is, from 203.7 ± 29.7 to 78.7 ± 14.2 pmol min^–1^ norm^–1^ (OCR) and from 51.8 ± 0.8.0 to 19.4 ± 3.5 pH min^–1^ norm^–1^ (ECAR), where norm denotes normalization to the cell count in the control (Figure 2d). This outweighs any effect MJ040X yields on mitochondrial metabolism. Hence, combining this with intracellular thermometry measurements, we conclude the main heating effect seen in cells with added Aβ42 derives from the exothermic effects of amyloid elongation rather than from mitochondrial damage, the latter of which is present but does not affect thermogenesis in our case.

### Heat Retention by a Protein is Enhanced by Ionic Interactions and Fewer Hydrogen Bonding Interactions with Water

We have thus far shown experimentally that the non-equilibrium effects of Aβ42 elongation in a cell leads to an increase in its overall temperature. Our results suggest the importance of these effects in understanding the microscopic mechanisms associated with amyloid aggregation. Because it is currently impossible to model the nonequilibrium conditions in the cell, we turned to using empirical-based classical molecular dynamics to examine some factors (e.g., protein–water hydrogen bonding and ionic interactions) that may contribute to why some released heat may reside longer in certain areas of the cells and potentially lead to the observed thermogenesis effect. We thus investigated whether (a) different amyloid structures or (b) the local intracellular environment may favor the retention of heat in the system.

**Figure 3:**
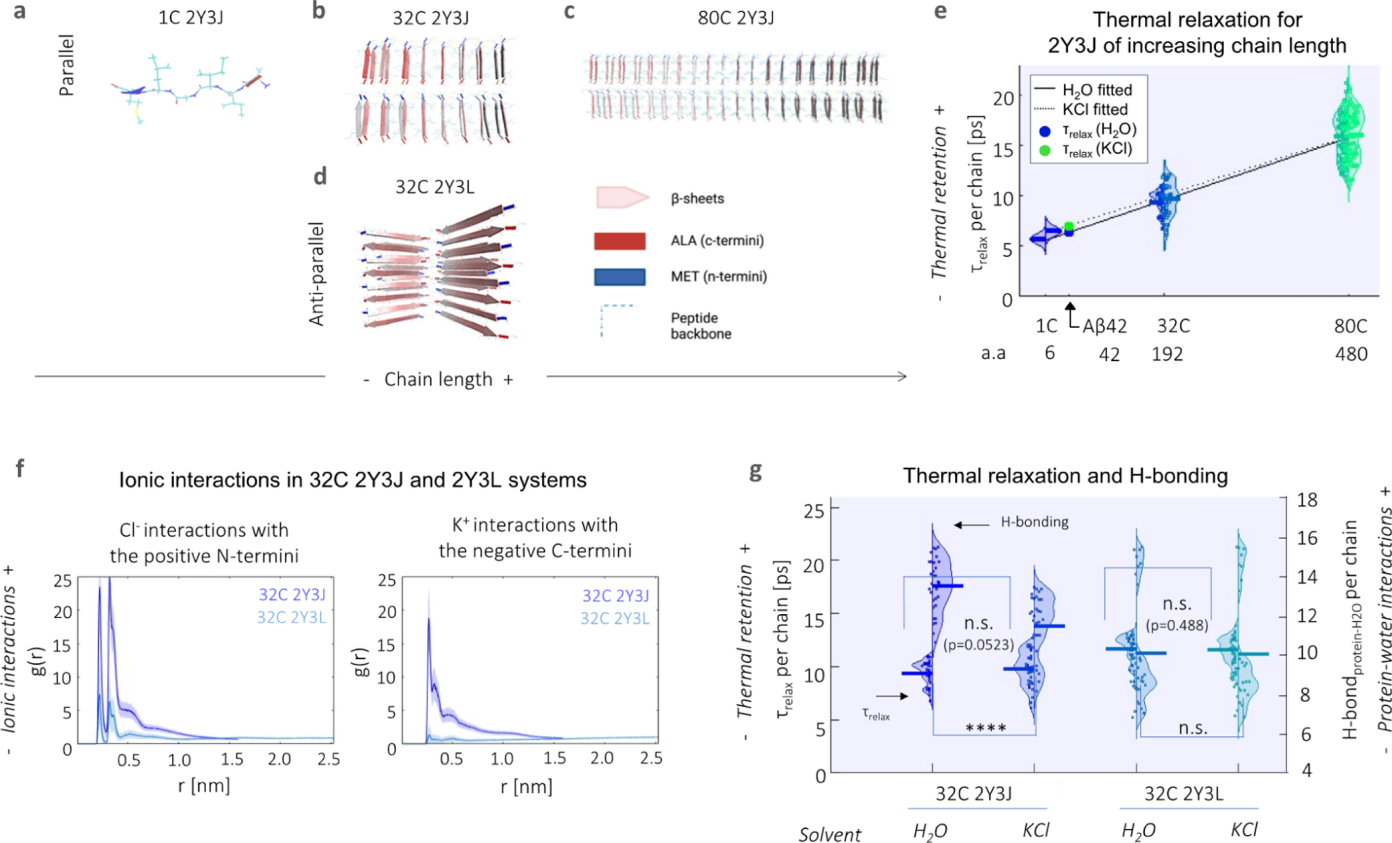
Heat retention by different Aβ structures is favored by fewer protein–water hydrogen bond interactions and in the presence of an ionic solvent. Structural illustration of model Aβ systems used, which include (a–c) parallel 2Y3J in (a) 1, (b) 32, and (c) 80 C; as well as (d) 32 C anti-parallel 2Y3L. (e) Relaxation times (τ_relax_) at the single chain level increase with larger structures of 2Y3J, following a linear trend. For Aβ, predictions for τ_relax_ show heat retention is slightly enhanced in KCl (7.05 ps, green dot) compared to H_2_O (6.38 ps, blue dot). a.a. denotes the number of amino acids. (f) Pair-correlation function [*g*(*r*)] shows that there is reduced ionic interaction with the charged terminal groups in anti-parallel 2Y3L, in comparison to parallel 2Y3J. (g) There is greater difference in both thermal retention (primary *y*-axis) and protein–water hydrogen bonding (secondary *y*-axis) between H_2_O and KCl in parallel 2Y3J than anti-parallel 2Y3L. *t*-test and one-way ANOVA tests (with Holm–Sidak’s multiple comparisons) are performed, where n.s. is not significant, ** is *p* < 0.005, *** is *p* < 0.001, and **** is *p* < 0.0001.

Aβ42 (similar to many misfolding proteins) possesses an intrinsically disordered nature, and the configuration which it adopts is influenced by ionic interactions with C- and N-termini and by wide networks of hydrogen bonding between protein and water.^23,24^ To simplify the case at hand, we first constructed molecular models based on different polymorphic segments of Aβ and investigate the effects of increased fibrillar size on heat dissipation. We started from the base Aβ crystal structure of 2Y3J (Aβ30-35)^25^ and 2Y3L (Aβ38-42),^25^ which differ in the arrangement of their like-charged terminal groups; hence, three-dimensional packing and arrangement of β-sheets: 2Y3J has a parallel β-sheet structure with like-charged terminal groups on the same side, while 2Y3L has an anti-parallel structure with the terminal groups alternating on each side. We concatenated them into separate 32 chain (32 C) structures (Figure 3b,d). To represent Aβ at different stages of elongation, we further expanded our 2Y3J model to include single chain (1 C, i.e., before the formation of β-sheets, Figure 3a) and 80 chain (80 C, i.e., upon further elongation, Figure 3c) structures. They are then submerged in four-site transferable intermolecular potential (TIP4P) water^26^ with and without the addition of 140 mM potassium chloride (KCl), the latter as proxy to an intracellular environment. Under the application of an all-atom optimized potential for liquid simulations (OPLS/AA) force field,^27^ the system is energy minimized, followed by equilibration to a canonical ensemble (*NVT*) using two separate Nose–Hoover (N–H) thermostats^28,29^ on the protein and water at 300 K. Heating of the protein to 400 K is then performed using the N–H thermostat, while the equivalent on water is kept at 300 K. The N–H thermostat of the protein is removed (whereas that of water is kept at 300 K) for production runs of thermal relaxation of the protein for 200 ps at a time step of 0.2 fs. Note, raising the temperature on the protein and the subsequent removal of its N–H thermostat is to measure heat dissipation in order to test if heat may reside longer in different Aβ structures when subject to different environmental conditions, and thereby contribute to the thermogenesis effect.

Rajabpour et al.^30^ have compared the modeling of solid–liquid interfaces, in their case, a silver nanoparticle–water system using different molecular dynamics approaches. They found that heat dissipation occurs at a time scale of ∼5 ps and that conduction is the primary heat transfer mechanism in the nanoparticle surroundings at this time scale. Hamzi et al.^31^ have also recently applied a similar protocol to study heat relaxation in the 20 naturally amino acids as well as in lysozyme protein. Interestingly, they find that heat dissipation is affected by both the size of the organic molecule and the underlying chemistry. As the Aβ crystal structures used are on the same length scale as a nanoparticle, we adopted a similar approach to study protein–water interfacial heat transfer and quantified relaxation time (τ_relax_, i.e., a measure of how quickly heat is dissipated from the protein, as calculated from exponentially decaying thermal relaxation curves) for each single chain comprising our model systems. We observe that single chain τ_relax_ increases with the chain length of the concatenated 2Y3J system, that is, from 1 to 32 and 80 C (Figure 3e). We perform linear fitting for 2Y3J in both H_2_O (solid line) and KCl solvents (dotted line) and note that chain length seems to outweigh heat dissipation differences due to solvent effects especially at longer chain lengths in our model systems. For Aβ42 [which has 42 amino acids (a.a.)], we predict a single chain τ_relax_ of 6.38 ps in H_2_O (blue dot) and 7.05 ps in KCl (green dot), by interpolation of the linear fits. Alongside this, we also observe that there is slightly lower diffusivity of water molecules in the hydration shell in the 80 C (0.33 ± 0.01 in water and 0.32 ± 0.02 × 10^–5^ cm^–2^ s^–1^ in KCl solvent) compared to 32 C structures (0.51 ± 0.09 in water and 0.37 ± 0.08 × 10^–5^ cm^–2^ s^–1^ in KCl solvent), which is in itself already hindered when compared to the diffusivity of bulk water molecules (∼2.8 × 10^–5^ cm^–2^ s^–1^) (Figure S6).

We next investigate the effects of ionic interactions both on heat dissipation and protein–water hydrogen bonding behavior using 32 C structures of parallel 2Y3J and anti-parallel 2Y3L in KCl ionic solution. By computing cross-correlation functions [*g*(*r*)] of K^+^ and Cl^–^ ions with the negative C- and positive N-termini, respectively, we observe that there are significantly lower ionic interactions in the case of 2Y3L, which is due to the weaker charge distribution across its structure (Figure 3f). Consequently, the slight increase in τ_relax_ (*p* = 0.0523) and decrease in protein–water hydrogen bonding for 2Y3J in KCl compared to in H_2_O, is not noticeable in the case of τ_relax_ (*p* = 0.488) and hydrogen bonding for 2Y3L (Figure 3g). Hence, this demonstrates that heat dissipation from proteins is affected by ionic interactions, as well as hydrogen bonding interactions between protein and water. Moreover, by tracking the gyration radius of the molecules during the thermal relaxation process, the 32 C 2Y3J takes on a more compact structure in KCl solution compared to water, giving a significant shrinkage in the gyration radius of 0.2 nm (Figure S7). This is analogous to the experimental observation of proteins having more globular conformations (i.e., more aggregated structures) in the intracellular environment.^32^ However, this effect is less pronounced for 32 C 2Y3L which we established has much weaker ionic interactions. Thus, thermal dissipation is hindered by increases in the Aβ42 fibrillar structure and ionic interactions.

## Discussion

Aβ42 aggregation leads to thermogenesis which may impact cellular function. Although the presence of Aβ42 alone compromises mitochondrial health, we find that this does not result in mitochondrial thermogenesis sufficient to be detected by intracellular thermometry measurements, in contrast to when FCCP was used. We show, for the first time in live cells, that Aβ42 elongation is directly responsible for elevating cell-averaged temperatures. This could not only enhance the elongation of Aβ42 further but also overcome the energy barrier for subsequent nucleation reactions, leading to the potential formation of oligomeric species which are more strongly associated with Aβ42-related pathology.^33^ These newly formed structures could further enhance the thermogenesis effect as they act as seeds for elongation.

Previous studies have revealed disease-related thermogenesis in cancerous cells^34^ and infected lesions^35^ using microcalorimetry measurements. On the other spectrum, it is interesting to note that hypothermia has been discussed as a potential neuroprotective therapy against dementia.^36,37^ Although the exact mechanisms are still unknown, the overexpression of a cold-shock protein, RNA binding motif 3 (RBM3), results in reduced synaptic and neuronal loss in mouse models of neurodegeneration.

Moreover, the FPT–FLIM can also be used as a drug screening platform, as measured temperatures were sensitive to both our positive control (i.e., FCCP which triggers severe mitochondrial damage and thermogenesis) and negative control (i.e., MJ040 which inhibits Aβ42 aggregation). This method is particularly advantageous as it does not require any fluorescent labels to be attached to the amyloid protein which may hamper aggregation kinetics^38^ and/or affect the formation of different Aβ42 polymorphs.^39^ The study further highlights the potential of small molecule drugs such as MJ040. Because MJ040 is a potent inhibitor of Aβ42 elongation, it might be administered at later stages of the disease and thereby block further nucleation processes by reducing Aβ42 elongation-induced thermogenesis. This is strongly supported by in vitro studies which show that small temperature increases significantly enhance Aβ42 nucleation.^40,41^

The main detraction of reported intracellular thermometry measurements is the large temperature gradients, which over exceed predicted values from the heat-diffusion equation by a factor of 10^5^.^42^ One of the most controversial reports include Chrétien et al.^43^ who, using MitoThermoYellow (a fluorescence-based mitochondrial-localised thermoprobe), observed that mitochondrial temperature is elevated up to 10 °C at its most active phase, without the use of any proton uncouplers. Nakano et al.^8^ measured a 6–9 °C temperature increase across the whole cell by the addition of FCCP using a ratiometric thermoprobe (genetically encoded ratiometric fluorescent temperature indicator, gTEMP) transfected into HeLa cells; this agrees with the current work which has seen a ∼10 °C increase in temperature with FCCP treatment in a different mammalian cell line, HEK293T. However, a similar study performed by Sugimura et al.,^44^ by exploiting the temperature-sensitive OH stretching band of water using Raman spectroscopy, saw a more modest increase of 1.8 °C (and only in the cytosol) after FCCP treatment. Discrepancies in reported temperatures between different groups could arise due to variations in thermoprobe localization, methods of performing the temperature calibration, experimental setup and methodology, and/or variations in cell cultures. In order to control for the latter, we performed the temperature calibration directly in the cells that were later used in the study, thus the sensor was already calibrated against potential cell to cell variability.

Semi-empirical models incorporating recent experimental findings made possible by advancement in the development of nano-/microscale thermoprobes are useful for understanding the highly non-equilibrium environment inside a biological cell. To understand our experimental findings, that is, the Aβ42 elongation associated cellular thermogenesis, we investigated, using classical molecular dynamics simulation, how different Aβ42 structures and the ionic intracellular environment could promote heat retention. Proteins have thermal conductivities 3 to 6 times smaller than that of bulk water; in other words, they can sustain larger temperature gradients across their bodies.^45^ By creating model Aβ peptides of different chain length to mimic Aβ at different stages of elongation, we show that larger structures form fewer hydrogen bonds with water at a single chain level and heat dissipation occurs at a slower rate.

On the other hand, ionic interactions lead to amyloid proteins adopting a more globular configuration and are surrounded by hydration shells within which dynamic properties are distinct from bulk water. Here, retarded water mobility around large aggregates could thereby trigger the formation of potential hotspots. The hydrophobic effect also comes into play, as crowding effects effectively promote the overlap of hydration shells belonging to two different proteins. The latter has been shown to result in the reduction to the overall hydration of the molecule and thereby triggers further aggregation.^32,46^ We further show that enhanced thermal retention in the presence of intracellular environment-mimicking KCl solution compared to water alone is due to ionic interactions and a decrease in protein–water hydrogen bonding interactions. However, further investigation (entailing non-equilibrium modeling) would be required to pinpoint the exact mechanisms which take place in an intracellular environment, where there is considerably higher packing density of proteins and other biomolecules.

## Conclusions

We show that Aβ42 elongation and not mitochondrial stress associated with Aβ42 aggregation is responsible for cellular thermogenesis as measured by FPTs, a highly sensitive temperature-dependent fluorescent sensor. We further validate that intracellular thermometry can be used as a tool to study Aβ42 aggregation and efficacy of an anti-aggregation small compound drug in a live cell model, thereby highlighting the potential of MJ040 as a therapeutic drug in AD. We turn to classical molecular dynamics to model different structures of Aβ in an attempt to shed some light onto the mechanisms behind the non-equilibrium effects that give rise to heat dissipation from amyloids. We show that this is affected by the structural nature of the amyloid peptides, the ionic strength of its surrounding environment (highly prevalent to an intracellular environment), and the extent of hydrogen-bonding interactions of the amyloid with water. With the model above, we hope to spark off further work in this field to develop more accurate descriptors of complex intracellular phenomena that give rise to increased temperatures within a biological cell, which can be indicative of neurodegeneration.

## Supporting Information Available

Methodology and additional experimental details, including: dSTORM imaging of immunostained Aβ42 in fixed cells; FLIM aggregation assay on HL488-labelled Aβ42 in live cells; in vitro ThT and AFM measurements on the effects of MJ040 on Aβ42; temperature to fluorescence lifetime calibration of FPT-FLIM; fluorescence emission decay profiles of ATeam1.03; effective diffusivity values; gyration radius traces; and comparison of FPT-FLIM data (PDF)

## Notes

Raw data is available through the Cambridge Apollo Repository DOI: 10.17863/CAM.82938.

## Abbreviations

Aβ: amyloid-β
AD: Alzheimer’s disease
dSTORM: direct stochastic optical reconstruction microscopy
ECAR: extracellular acidification rates
ETC: electron transport chain
FLIM: fluorescence lifetime imaging microscopy
FPTs: fluorescent polymeric thermometers
FRET: Förster resonance energy transfer
OCR: oxygen consumption rates
TCSPC: time-correlated single photon counting
