# Supplementary material for: Intracellular Aβ42 Aggregation Leads to Cellular Thermogenesis
Source: J Am Chem Soc. 2022 May 26;144(22):10034–41. doi: 10.1021/jacs.2c03599 (PMC9185738; doi:10.1021/jacs.2c03599)
Supplement: Supplementary file 1 — ja2c03599_si_001.pdf [file ja2c03599_si_001.pdf]

Supporting Information

Intracellular Aβ42 aggregation leads to cellular thermogenesis

Chyi Wei Chung[a], Amberley D. Stephens[a], Tasuku Konno[b], Edward Ward[a], Edward Avezov[b], Clemens F. Kaminski[a], Ali A. Hassanali[c] and Gabriele S. Kaminski Schierle[a*]

[a] Department of Chemical Engineering and Biotechnology, Philippa Fawcett Drive, University of Cambridge, Cambridge, CB3 0AS, UK
[b] UK Dementia Research Institute, Department of Clinical Neuroscience, University of Cambridge, Cambridge, CB2 0AH, UK
[c] Condensed Matter and Statistical Physics, International Centre for Theoretical Physics, Strada Costiera 11, Trieste 34151, Italy
[*] Corresponding author: gsk20@cam.ac.uk

Table of Contents

Methods *1*

Cell culture 1

AβM1-42 Purification 1

Fixing of cells for the immunostaining of Aβ42 2

dSTORM 3

ThT-based aggregation assays of recombinant Aβ42 4

Atomic force microscopy on Aβ42 5

TCSPC-FLIM 5

FPT-FLIM 6

ATeam1.03 ATP FRET sensor 7

Seahorse Mito Stress Assay 8

Molecular dynamics simulations 8

Statistical analysis and plotting 9

Supplementary figures *10*

Supplementary Figure 1: Exogenously added monomeric WT-Aβ42 forms fibrillar aggregates in HEK239T. 10

Supplementary Figure 2: MJ040X, a small molecule drug, reduces the extent of Aβ42 aggregation. 12

Supplementary Figure 3: MJ040 significantly inhibits the elongation of Aβ42 in vitro. 13

Supplementary Figure 4: Temperature calibration of FPTs in live cells reveals a temperature resolution of 14

Supplementary Figure 5: Representative fluorescence emission decay profiles of the ATP sensor ATeam1.03. 16

Supplementary Figure 6: Large aggregates are more likely to promote heat retention. 17

Supplementary Figure 7: 32C 2Y3J is more aggregated in KCl than in water. 18

Supplementary table *19*

Supplementary Table 1: Comparison of FPT-FLIM data where fluorescence lifetimes are quoted as mean of bi-exponential lifetimes (exponential fitting) and modulation lifetimes (phasor plot) 19

References *20*

Methods

Cell culture

Human embryonic kidney cells (HEK293T, American Type Culture Collection (ATCC), Manassas, VA, USA), cultured in a T75 cell flask with media, were incubated at 37 °C and 5% CO_2. Media comprised of 90 v/v% Dulbecco's modified Eagle's medium (DMEM, ThermoFisher Scientific, Waltham, MA, USA), 10 v/v% fetal bovine serum (FBS, ThermoFisher Scientific), and 2 mM each of glutamax (ThermoFisher Scientific) and 2% penicillin-streptomycin (ThermoFisher Scientific). HEK293T were passaged when 80–90% confluency was reached (i.e., twice a week). HEK293T cells were plated into 8 well plates (IBIDI GmbH, Gräfelfing, Germany), and grown until 40—70% confluency before FPT introduction. For the cells with Aβ42, 500 nM solution of unlabelled Aβ42 monomer was added. For cells with MJ040X, 2.5 µM from a 100/200 µM stock in DMSO (Life Technologies, ThermoFisher Scientific) was added. Both Aβ42 and MJ040X were incubated for 24 hours before imaging. FCCP (Merck KGaA, Darmstadt, Germany) treatment was performed during the day of imaging, with FCCP added at a concentration of 10 µM (from a 10 mM stock in DMSO) and cells were left to incubate for 30 minutes. All reagents were kept at -20 °C and aliquoted to prevent freeze-thaw cycles.

AβM1-42 Purification

Recombinant AβM1-42 (also referred to as Aβ) was purified as described in Stephens *et al.* [1]. The plasmid pET3a containing AβM42 cDNA was transformed into *Escherichia coli* (*E. coli*) One Shot BL21 (DE3) pLysS (ThermoFisher Scientific). Liquid culture of *E. coli* was induced for expression for 4 hours when the OD_{600} reached 0.6–0.8 by the addition of 1 mM isopropyl-β-thiogalactopyranoside (IPTG). The cells were pelleted in 50 mL volumes and the supernatant discarded. The pellet contained Aβ in inclusion bodies. The inclusion bodies were washed four times in wash buffer, as detailed in [1] to obtain clean and pure inclusion bodies. The inclusion body pellet from 50 mL of culture was placed on ice with

a small magnetic stir bar on a magnetic stirrer. 200 µL of 6 M guanidinium chloride (GuHCl) was added to the pellet and stirred vigorously for 30 min to solubilise the Aβ. 15 mL of ice-cold ion exchange (IEX) buffer A (10 mM Tris, 1 mM ethylenediaminetetraacetic acid (EDTA), pH 9) was added slowly to the solubilised pellet to dilute the 6 M GuHCl and to permit binding of Aβ to the ion exchange column. The solubilised Aβ was filtered through a 0.22 µm filter (Millex-GP, MillPore, Merck KGaA) before being placed on ice prior to chromatography. Aβ was loaded onto a HiTrap Q HP column (GE, Healthcare, Chicago, IL, USA) and eluted against a linear gradient of IEX buffer B (10 mM Tris, 1 mM EDTA, 0.75 M NaCl, pH 9) over seven column volumes followed by two column volumes of 100% buffer B. Purification was performed on an ÄKTA Pure Fast Protein Liquid Chromatography (FPLC) and monitored by absorption at 280 nm (GE Healthcare). The concentration of Aβ was determined by absorption at 280 nm on a NanoVue spectrometer (Biochrom Ltd., Cambridge, UK) using the extinction coefficient of 1490 M^{-1} cm^{-1}.

Fixing of cells for the immunostaining of Aβ42

Cell media was removed and replaced with 4% paraformaldehyde (PFA, Merck KGaA) diluted in 1×PBS. The sample was fixed for 10 minutes, before blocking with 5 w/v% bovine serum albumin (BSA, Merck KGaA) diluted in PBS for 1 hour. Between antibody incubation, three washes of 50 µM Triton X-100 (Invitrogen, ThermoFisher Scientific) in PBS (henceforth referred to as PBST) were performed. Primary and secondary antibodies used were β-Amyloid(1-42) polyclonal antibody (Invitrogen, ThermoFisher Scientific) and Goat anti-Rabbit Alexa Fluor 647 (ThermoFisher Scientific), diluted by 1:100 and 1:400 in PBST respectively. The sample was kept at room temperature throughout all the steps and wrapped in aluminium foil to prevent any bleaching especially after the addition of the secondary antibody. For storage before imaging, the sample was kept at 4 ^{o}C in dark conditions.

d*STORM*

Before imaging the fixed cells, PBS is replaced with *d*STORM photo-switching buffer consisting of 50 mM Tris pH8 solution supplemented with 10 mM sodium chloride (ThermoFisher Scientific), 10% glucose (ThermoFisher Scientific), 50 mM monoethanolamine (MEA, Merck KGaA), 0.5 mg/mL glucose oxidase (Merck KGaA) and 40 µg/mL catalase (Merck KGaA). They were mounted on a custom-build microscope with an IX-73 Olympus frame (Olympus) with a 647 nm laser (VFL-P-300-647-OEM1-B1, MPB Communications Inc., Quebec, Canada). Laser light entering the microscope frame was reflected from a dichroic mirror (ZT647rpc, Chroma, Bellows Fall, VT, USA) onto a 100X 1.49 NA oil total internal reflection (TIRF) objective lens (UAPON100XOTIRF, Olympus), before reaching the sample. Light emitted by the sample passed through the dichroic and a set of 25 mm band-pass filters (FF01-680/42-25, Semrock) before reaching the microscope side port. Images were then relayed onto a camera (Andor iXon Ultra 897, Oxford Instruments, Belfast, UK) by a 1.3x magnification Twincam image (Cairn, Kent, UK). The image pixel size was measured to be 117 nm using a ruled slide. Each 256x256 image was acquired as stacks of 15000 images with an exposure time of 0.01 ms. Around eight images were captured per biological repeat. Fluorophore localisations are first detected using the Fiji plugin, ThunderSTORM[2], before reconstruction using an in-house MATLAB script detailed in the methodology section of [3]. Reconstructed images were then further analysed in a separate MATLAB script to quantify the major axis length (i.e., longest dimension) and eccentricity (Equation 1) of Aβ42 aggregates.

Equation 1

$$Eccentricity = \sqrt{1 - \frac{b^2}{a^2}}$$

where, a and b are the semi-major and semi-minor axes of an ellipse, respectively. Aggregate masking was achieved by an intensity threshold, following a pixel-size threshold to ensure that only

aggregates with dimensions above the resolution limit (i.e., estimated at 70 nm) were included in the analysis.

ThT-based aggregation assays of recombinant Aβ42

20 µM of freshly made thioflavin-T (ThT) (abcam, Cambridge, UK) was added to 10 µM Aβ in 170 mM NaCl, 30 mM Tris, pH 7. 50 µM of MJ040 or the equivalent volume of DMSO, which MJ040 is dissolved in, were added. 10% seeds were made by incubating 10 µM of Aβ for 24 hours and sonicating them for 5 seconds at 30% amplitude (Digital Sonifier® SLPe, model 4C15, Branson, Danbury, MA, USA). 25 µL of each sample was added in triplicate to a 368-well plate black plate with a clear bottom (Greiner Bio-One GmbH). The plates were sealed with a SILVERseal aluminum microplate sealer (Greiner Bio-One GmbH). Fluorescence measurements were taken using a FLUOstar Omega plate reader (BMG LABTECH GmbH, Ortenbery, Germany). The plates were incubated at 37 °C with double orbital shaking at 300 rpm for 15 seconds before each read every 10 minutes for 8 hours. Excitation was set at 440 nm, and the ThT fluorescence intensity was measured at 480 nm emission with a 2200 gain setting. ThT-based assays were repeated three times and the data normalized to the maximum fluorescence per plate. A linear trend line was fitted along the exponential phase of the ThT fluorescence curve (shown in **Supplementary Figure 3**) and **Equation 2** was used to calculate the time to form the first fibrillary structures, lag time (t_{lag}), at the intercept of the x axis, and the elongation rate from the slope of the exponential phase (k), indicating growth rate.

Equation 2

$$y = kx - t_{lag}$$

Atomic force microscopy on Aβ42

The contents of wells from the ThT-based assays were deposited on a freshly cleaved mica surface for 20 min. The mica was washed three times in 18.2 MΩ.cm dH_2O to remove loose protein. Images were acquired in dH_2O using tapping mode on a BioScope Resolve (Bruker GmbH, Karlsruhe, Germany) using 'ScanAsyst-Fluid+' probes. 256 lines were acquired at a scan rate of 0.966 Hz per image with a field of view of 4 µm. Images were adjusted for contrast and exported from NanoScope Analysis 8.2 software (Bruker GmbH).

TCSPC-FLIM

Samples were imaged on a home-built confocal fluorescence microscope equipped with a time-correlated single photon counting (TCSPC) module. A pulsed, supercontinuum laser (Fianium Whitelase, NKT Photonics, Copenhagen, Denmark) provided excitation a repetition rate of 20 MHz (for FPT-FLIM) and 40 MHz (for imaging ATeam1.03 FRET sensor). This was passed into a commercial microscope frame (IX83, Olympus, Tokyo, Japan) through a 60x oil objective (PlanApo 60XOSC2, 1.4 NA, Olympus). The excitation and emission beams are filtered through select filters (Semrock, Rochester, NY, USA): (i) FF01-474/27-25 & FF01-515/LP-25 for FPTs and (ii) FF01-434/17 & FF01-470/28 for ATeam1.03. Laser scanning was performed using a galvanometric mirror system (Quadscanner, Aberrior, Gottingen, Germany). Emission photons were collected on a photomultiplier tube (PMT, PMC150, B&H GmbH, Berlin, Germany) and relayed to a time-corelated single photon counting card (SPC830, B&H GmbH). Images were acquired at 256x256 pixels for 200 s (i.e., 20 cycles of 10 s). Photon counts were kept below 1% of laser emission photon (i.e., SYNC) rates to prevent photon pile-up. TCSPC images were analysed using an in-house phasor plot analysis script (https://github.com/LAG-MNG-CambridgeUniversity/TCSPCPhasor), from which fluorescence lifetime maps and phasor plots were generated.

FPT-FLIM

The linear cationic FPT (Funakoshi, Tokyo, Japan) was soaked overnight in Milli-Q water (Merck Millpore, Merck KGaA) to permit the full extension of the polymer, forming a 1 w/v% stock solution. On the day of imaging, the FPT stock solution was diluted in 5 w/v% glucose solution (made from a stock of 45% D-(+) glucose solution, Merck KGaA), to give a concentration of 0.03 w/v% FPT. Cell media was removed from a plated well and washed twice with 200 µL 1xPBS (ThermoFisher Scientific). 150 µL of 0.03 w/v% FPT solution was added to each cell well. After incubation at 25 °C without CO_2 for 30 minutes, unincorporated FPT solution was removed, and each well was washed twice with 150 µL phosphate buffer solution (1×PBS). 250 µL of phenol red free Dulbecco's Modified Eagle's Medium (DMEM) (ThermoFisher Scientific), supplemented with 10% fetal bovine serum (FBS), was then added to the cell wells, ready for imaging.

Due to the relatively low fluorescence intensity of the FPT, a long-pass filter was used to maximise collection. The stage top heater (OKOLab, Ottaviano, Italy) was set at 30 °C, as the FPT are noted to clump at 37 °C; and at 5% CO_2 and 20% relative humidity. An objective warmer (OW-1D, Multi-Channel Systems (MCS) GmbH, Reutlingen, Germany) was used to avoid the heat sink effect resulting from the significantly different thermal conductivities of the oil and bottom glass surface of the cell dish. This is controlled by a single channel single controller (TC-324C, MCS GmbH), which included a thermocouple (TA-29, MCS GmbH) that was inserted into the cell media through a small hole drilled on the lid to ensure cell media temperature is maintained at 30 °C. Results are based on >30 cells over three biological repeats.

The imaging setup was first validated using Rhodamine B (Merck KGaA), a standard fluorescence dye with fluorescence lifetimes that decrease as temperature increase.[4] (see **Supplementary Figure 4a—c**) Calibration of temperature to fluorescence lifetime in live HEK293T was

performed by temperature stepping between 28—45 °C (assumed temperatures were based on readings from the thermocouple inserted in the cell media), by adjusting temperature settings on both the OKOLab stage-top heater and objective warmer system. The final calibration equation based on modulation lifetime (τ_M), in lieu of phase lifetime (τ_ϕ) due to its higher sensitivity to temperature, as FPT phasors move more horizontally across the phasor plot (**Supplementary Figure 4d**). For fluorescence lifetime, phasor plot analysis was applied instead of conventional exponential fitting, as the chosen is a fit-free non-statistical method, hence permits more accurate complex exponential decay analyses.[5,6] Temperature resolution (dT) is calculated using the same equation as in the original publications on the FPTs[7,8], and also as Equation 3:

Equation 3

$$dT = \frac{\partial T}{\partial \tau} d\tau$$

where $\frac{\partial T}{\partial \tau}$ is the reciprocal of the gradient in the fluorescence lifetime to temperature calibration, and $d\tau$ is the standard deviation of fluorescence lifetime at each given temperature. Using calculated modulation lifetime based on phasors generated, the system was found to have a temperature resolution of 0.74 °C, with modulation lifetimes ranging between 7.8 to 11.5 ns to cover a temperature range of 24 to 45 °C (see **Supplementary Table 1** for comparison to original publications, and **Supplementary Figure 4d—f**).

ATeam1.03 ATP FRET sensor

Design of the Förster resonance energy transfer (FRET-)based, cytosolic ATP sensor, ATeam1.03 can be found in Imamura *et al.*[9]. Two days before imaging, 200 ng ATeam1.03 plasmid diluted in antibiotics-free DMEM (ThermoFisher Scientific) was transfected using Lipofectamine 2000 (ThermoFisher Scientific) four hours after which the medium was changed, and the cells were kept incubated for another 20 hours before imaging. TCSPC-FLIM was only performed on the CFP donor channel whilst the

YFP channel was used as a guide to confirm that the fluorescence seen in the CFP channel is due to ATeam1.03 rather than cell autofluorescence. ATeam1.03-nD/nA/pcDNA3 was a gift from Takeharu Nagai (Addgene plasmid number 51958; RRID: Addgene_51958). Analysis for the ATeam1.03 imaging was performed on SPCImage (B&H GmbH) where reduced fluorescence lifetime of CFP was calculated by bi-exponential fitting.

Seahorse Mito Stress Assay

HEK293T were plated in 96 well plate (XF96, Agilent, Santa Clara, CA, USA), and 500 nM Aβ42 and/or 2.5 µM MJ040X were added alongside 24 hours before the Seahorse Assay. A Seahorse XFe96 analyser (Agilent) was used to perform the XF Cell Mito Stress Assay. Measurements had 18 mins intervals between drug addition, i.e., 1.5 µM oligomycin, followed by 1 µM FCCP and 0.5 µM rotenone/antimycin A (as part of Seahorse XF Cell Mito Stress Test Starter Pack, Agilent). Results are based on 3 biological repeats, with 5 wells plated per sample each time. Data were normalised to control according to cell count, as calculated by an automated cell counter (CountessII FL, ThermoFisher Scientific). Analysis was performed on Wave (Agilent, USA) before importing to MATLAB for plotting purposes.

Molecular dynamics simulations

GROMACS (v2021, DOI: https://doi.org/10.5281/zenodo.4457626) was used for all simulations, with structures for Aβ30-35 (2Y3J) and Aβ35-42 (2Y3L) taken from the protein database. For concatenation of 2Y3J/L, Mercury (Cambridge Crystallographic Data Centre (CDCC), University of Cambridge) was used to alter the protein database (pdb) file. Each model is generated by fully solvating the Aβ peptide in TIP4P water within a triclinic box, with box edges at least 1 nm away from the peptide to satisfy periodic boundary conditions. For the KCl solvent, the equivalent of 140 mM of K^+ and Cl^- ions were added, as all Aβ structures were of a neutral charge, the same number of cations and anions are added to preserve system neutrality. All systems were subject to an OPLS/AA forcefield[10] and energy

minimisation before being equilibration to a micromechanical (NVE) ensemble at 300 K for 100 ps. Upon comparison of popular forcefields used for Aβ peptide simulations, Smith *et al.*[11] showed that both OPLS/AA and GROMOS produce results which are most consistent with experimental data, as they supress the formation of helical structures. Heating of the peptide was achieved by introducing Nose-Hoover thermostat[12,13] at 400 K and 300 K on the peptide and water, respectively, for 20 ps to generate initial conditions. Simulations were performed to 200 ps at a timestep of 0.2 fs. Data output from simulations (in the form of .trr, .xvg) were then imported into MATLAB for batch analyses and producing figures and graphs. Relaxation time (τ_{relax}) is calculated by linearising the exponentially decaying temperature profile (Equation 4). Hydration shells are assumed as water molecules at 0.3 nm away from the peptide.

Equation 4

$$\ln \frac{T(t) - T_w}{T_i - T_w} = -\frac{t}{\tau_{relax}}$$

where T is temperature of the peptide at a given time, T_W is temperature of the water (300 K), T_i is temperature of the peptide at time zero (i.e., 400 K in most cases) and t is time. Thermal relaxation times and hydrogen bonding with water were calculated based on each chain within the peptide structure. Visualisation of the structures were produced using VMD.[14]

Statistical analysis and plotting

All statistical analyses were performed on Prism 6 (GraphPad, San Diego, CA, USA), where either a one-way ANOVA test (with Holm-Sidak's multiple comparison) or t-test was applied. Violin plots were produced using adapted, open-source MATLAB code from Anne Urai (github.com/anne-urai).

Supplementary figures

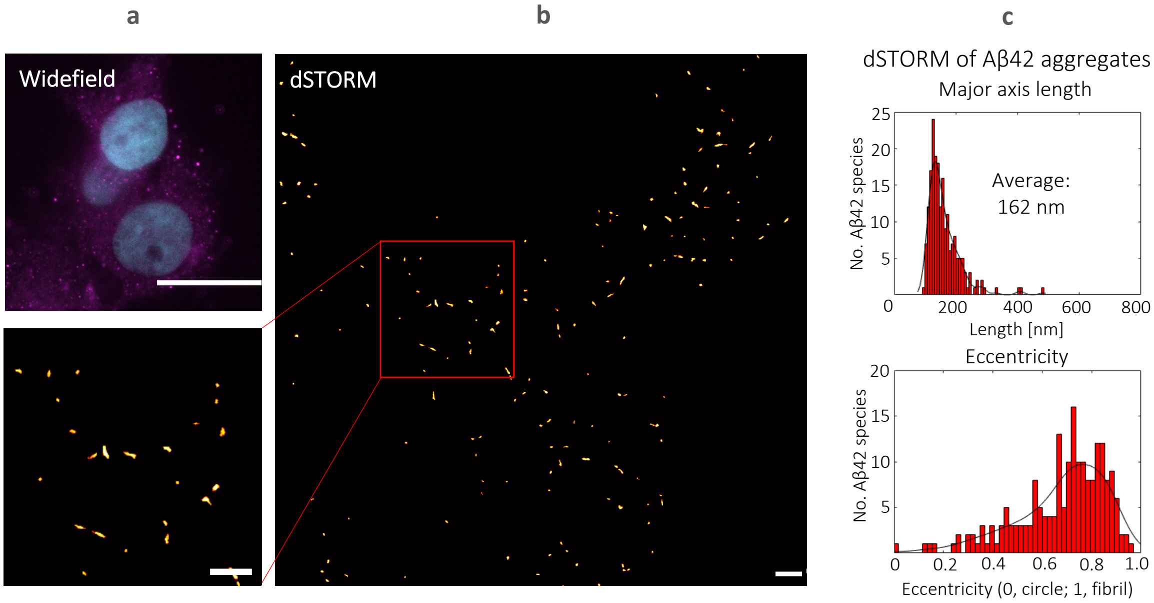

Supplementary Figure 1: Exogenously added monomeric WT-Aβ42 forms fibrillar aggregates in HEK239T. (a) Widefield imaging provides insufficient resolution, as AF647 tagged antibody labelled Aβ42 (magenta) has dimensions below the diffraction limit. Cell nuclei are additionally stained with Hoechst 33342 to highlight Aβ42 formed only in the cytoplasmic regions. Scale bar, 10 µm. (b) dSTORM images of AF647 tagged antibody labelled Aβ42 (red). Scale bars in main and zoomed image, 500 nm. (c) Morphological quantification in terms of major axis length (i.e., longest dimension length) and eccentricity (i.e., how fibrillar the structure is). 28 images were taken over 3 biological repeats.

We incubate HEK293T cells with monomeric, unlabelled WT-Aβ42 for 24 hours to allow sufficient time for aggregate formation. The use of unlabelled protein provides better physiological relevance (i.e., avoids steric hindrance associated with fluorescent tags) and avoids any cross talk with the relatively dim FPT signal. As subsequent thermometry experiments are performed without being able to visualise Aβ42, a homogenous distribution of Aβ42 throughout cells was desired. Upon fixing the cells and immunostaining for Aβ42, we perform super-resolution *direct* Stochastic Optical Reconstruction Microscopy (*d*STORM). The technique works by localising fluorescence signal of a photoactivatable fluorophore (i.e., Alexa Fluor 647 in this case) undergoing photo-switching over 15,000 frames captured at a high speed. Aβ42 aggregate structures formed appear primarily as elongated structures (i.e., with eccentricity values tending towards unity) with an average length of 162 nm, dispersed throughout the cytoplasmic region of the cells. Calculated dimensions agreed with values quoted by Esbjörner *et al.* [3],

who visualised intracellular HiLyte-647 tagged Aβ42 in SH-SY5Y cells, using the same imaging setup. However, it should be noted that the aggregates formed were significantly smaller than the equivalent in the presence of pre-formed seeds.[15] Moreover, in comparison to the more aggressive familial mutant, E22G-Aβ42, structures formed do not display the same degree of polymorphism (i.e., ranging from clusters to bundles and large perinuclear aggresomes).[16]

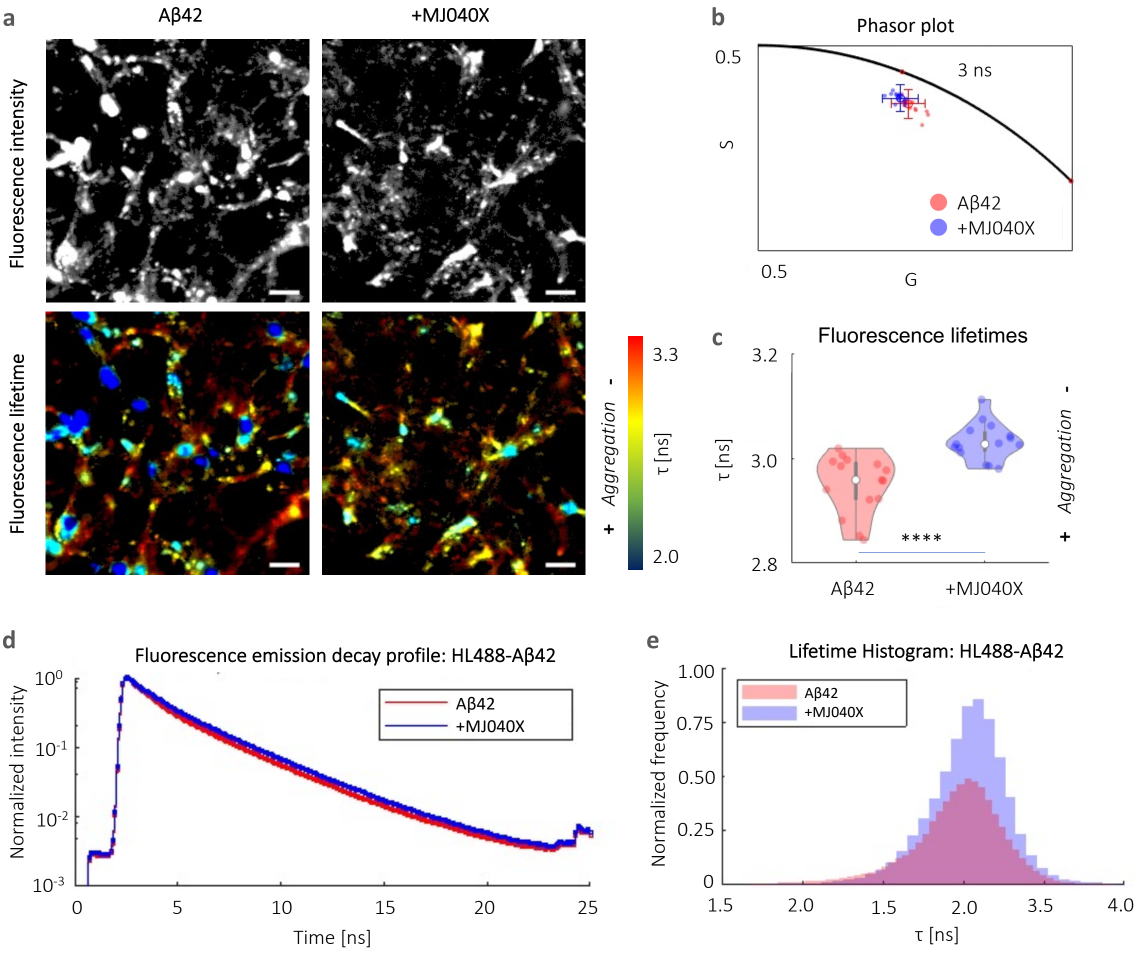

Supplementary Figure 2: MJ040X, a small molecule drug, reduces the extent of Aβ42 aggregation. Experiments are based on measuring the fluorescence lifetime of 10% HiLyte488 (HL488) tagged Aβ42 added to 90% unlabelled Aβ42. (a) Fluorescence lifetime maps, (b) phasors, and (c) averaged fluorescence lifetimes show that the addition of MJ040X alleviates Aβ42 aggregation. Based on 12 images collected over 3 biological repeats. Significance based on t-test, where **** is $p<0.0001$. (d) Fluorescence emission decay profiles of 10% HL488 tagged Aβ42. (e) Fluorescence lifetime of HL488tagged Aβ42 in the presence or absence of MJ040X.

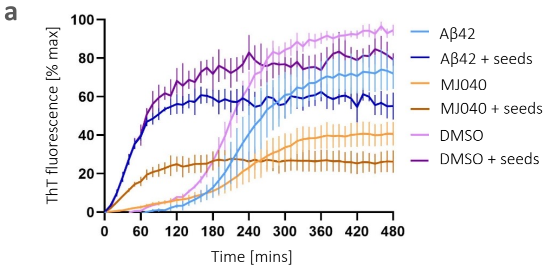

b

	t_{lag} [min]	k [min^{-1}]
Aβ	168.5 ± 20.5	0.52 ± 0.07
MJ040	154.1 ± 30.4	0.25 ± 0.02**
DMSO	138.6 ± 9.3	0.7 ± 0.02
Aβ + seeds		0.72 ± 0.05
MJ040 + seeds		0.29 ± 0.02*,*
DMSO + seeds		0.77 ± 0.06

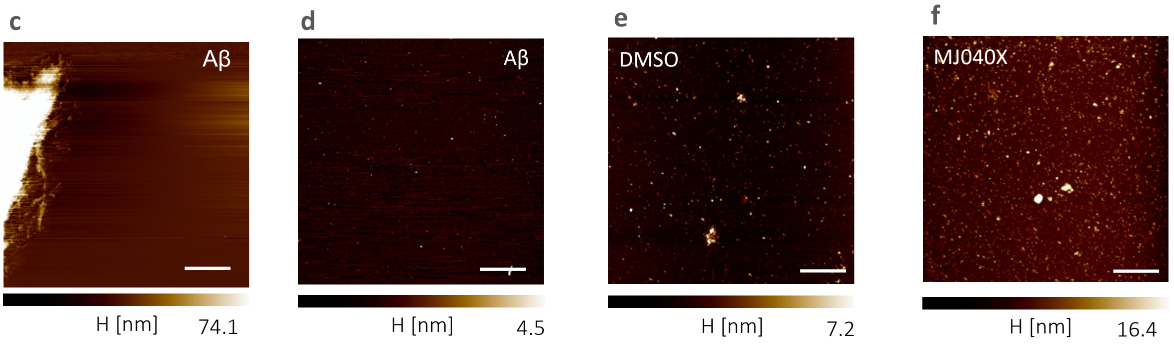

Supplementary Figure 3: MJ040 significantly inhibits the elongation of Aβ42 in vitro. MJ040 was used in ThT-based assays as this is the ester cleaved form of MJ040X that would be found in cells inhibiting Aβ42 aggregation. ThT-based aggregation assays show 50 µM MJ040 inhibits elongation of Aβ42 in the presence and absence of 10% Aβ42 seeds. (a) 10 µM Aβ42 in 170 mM NaCl, 30 mM Tris, pH 7 with 20 µM ThT in a 368-well plate was incubated at 37°C for 8 hours. ThT fluorescence intensity was measured every 10 minutes, with double orbital agitation at 300 rpm for 15 seconds before each read. The average ThT fluorescence is presented as a percentage of the maximum fluorescence measured per plate, which was determined by calculating the average of the fluorescence of three wells per condition. The experiment was repeated 3 times and the error bars represent s.e.m. The equivalent volume of DMSO (v/v) to MJ040 was added as a control. (b) The time to form fibrils (t_{lag}) and the elongation rate (k) were calculated from a linear fit to the exponential phase of the fibril growth curves from three individual experiments (Equation 2). MJ040 significantly inhibited the elongation rate, (* is p<0.025 compared to Aβ42 + seeds and DMSO + seeds and ** is p<0.0073 compared to v/v DMSO using a one-way ANOVA with Dunnet's multiple comparison test). (c—f) Representative AFM images show the morphology of the samples after ThT-based assays. (c) The Aβ42 only sample showed presence of fibril clusters, (d) but also the presence of small fibrils and oligomers. For (e) DMSO and (f) MJ040 only oligomers were detected.

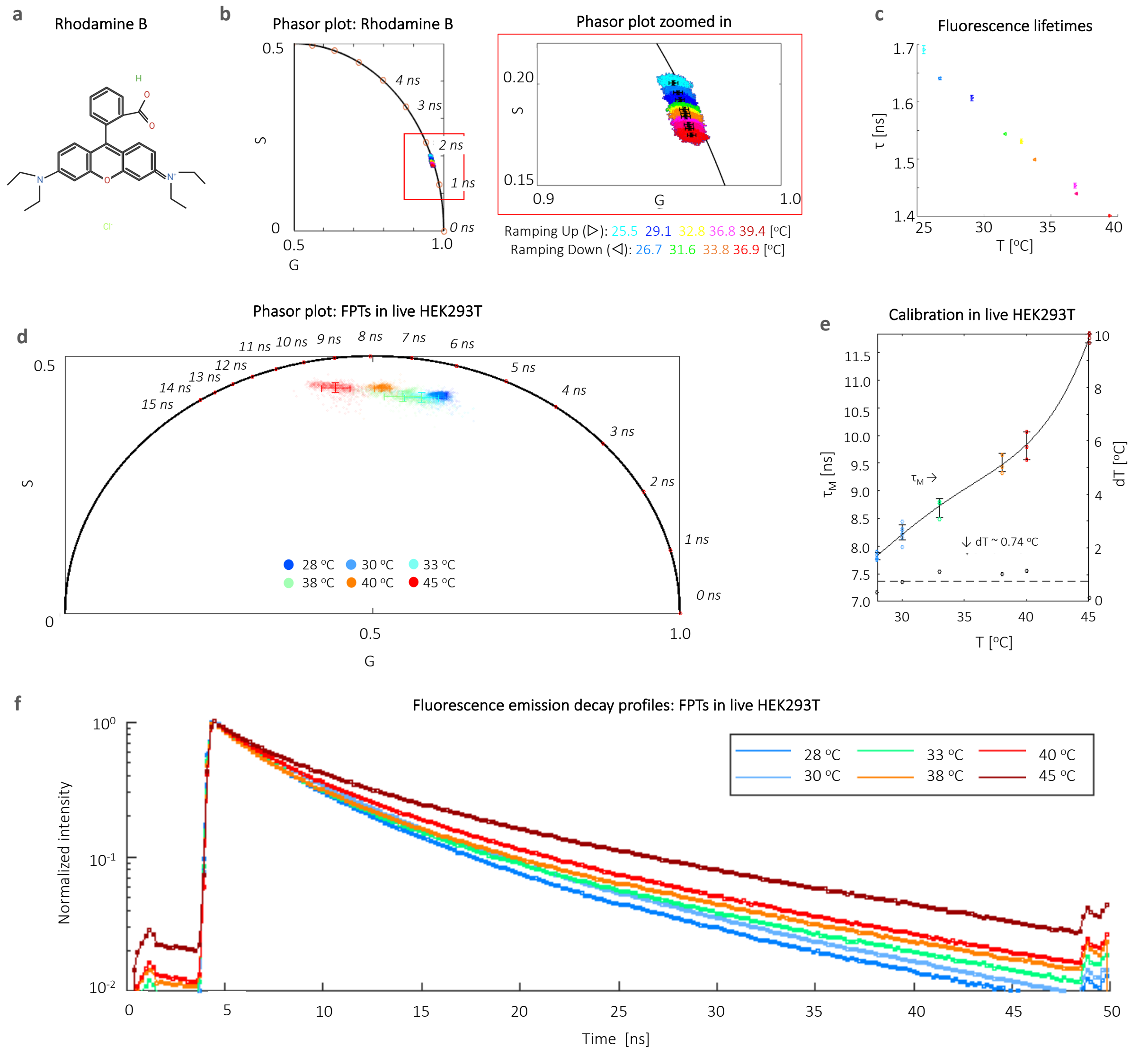

Supplementary Figure 4: Temperature calibration of FPTs in live cells reveals a temperature resolution of 0.74 °C. (a) Chemical structure of Rhodamine 6G. Created on Biorender.com, based on [17]. (b—c) The system was first validated with Rhodamine B, where a decrease of fluorescence lifetimes with increasing temperature was observed as seen in both (b) the phasor plot and (c) corresponding fluorescence lifetime values. (d) FPT-FLIM calibration shows a horizontal, anticlockwise trajectory of FPT phasors with increasing temperatures, hence (e) modulation lifetimes were used to calibrate the system to a temperature resolution of 0.74 °C (calculated using Equation 3) between 28—45 °C. (f) Fluorescence emission decay profiles of FPTs for FPT-FLIM temperature calibrations.

Temperature was determined by a thermocouple inserted into the cell medium, and an objective warmer (to prevent the heat sink effect by the objective) was used in conjunction with a stage top heater. To validate the ability of the system setup in terms of temperature stability and ramping, we first tested the system with Rhodamine B (**Supplementary Figure 4a**), a standard fluorescence dye that

has a temperature-dependent fluorescence lifetime readout due to changes in the mobility of its diethylamino groups[18]. Measured fluorescence lifetime was reduced from 1.7 ns at 25 °C to 1.4 ns at 45 °C, agreeing with values in the literature[4] (**Supplementary Figure 4b—c**). Calibration in live cells is considered the more valid method (**Supplementary Figure 4d—f**), as it mirrors the actual experimental setup, however it is more time-consuming and difficult to perform. Hence, in addition, calibration in a cell extract solution (recommended as the easier method by Inada *et al.*[7]) was also attempted; however, clumping of the dyes in the cell extract became visibly apparent above 35 °C and fluorescence lifetime readings using TCSPC-FLIM became unreliably low in values. Hence, it is believed that cell extract calibration is only feasible in a spectrophotometer, a method more sensitive than TCSPC-FLIM and that does not involve inverted imaging, where FPT clumps become unavoidable. A comparison to calibration performed by the original developers of the FPTs [7,8] is given in **Supplementary Table 1**.

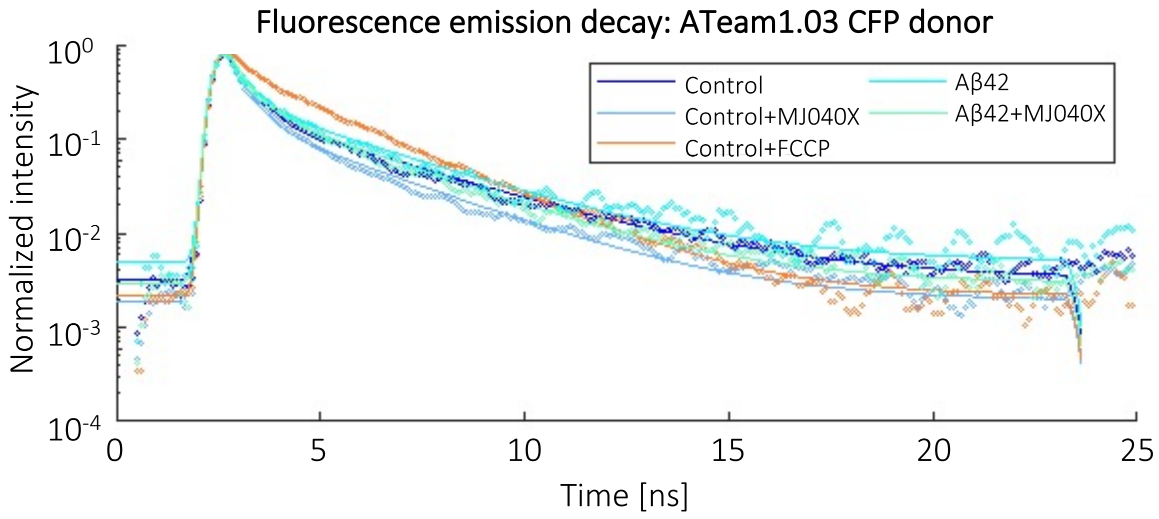

Supplementary Figure 5: Representative fluorescence emission decay profiles of the ATP sensor ATeam1.03. Non-linear decay profiles plotted on a logarithmic scale reflect the bi-exponential decay of the FRET donor, in this case CFP. Solid lines and scatters represent the bi-exponential fit model and raw traces, respectively.

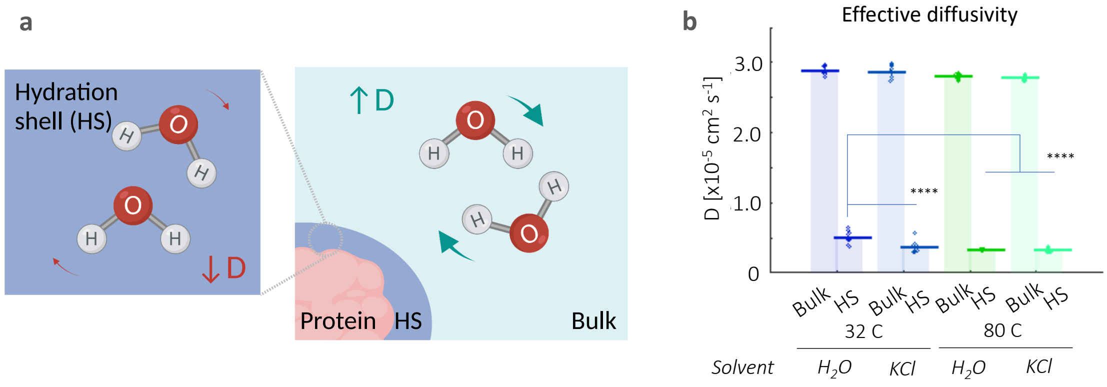

Supplementary Figure 6: Large aggregates are more likely to promote heat retention. (a) Water molecules in hydration shells (HS) that surround proteins submerged in solution have dynamic properties that differ from those in bulk water, e.g., lowered diffusivity in HS. Created on BioRender.com. (b) Effective diffusivity (D) values calculated from MSDs see up to a three-fold decrease in the HS compared to the bulk in both the 32 and 80C systems. There is a slight but significant effect of ionic KCl solution on hindering effective diffusivity in the 32 C system. Mean and standard deviation values of effective diffusivity are 2.89±0.05 (32C H_2O), 2.87±0.09 (32C KCl), 2.80±0.04 (80C H_2O), 2.78±0.03 x10^{-5} cm^{-2} s^{-1} (80C KCl) for the bulk; and 0.51±0.09 (32C H_2O), 0.37±0.08 (32C KCl), 0.33±0.01 (80C H_2O), 0.32±0.02 x10^{-5} cm^{-2} s^{-1} (80C KCl) for the HS. One-way ANOVA (Holm-Sidak's multiple comparison) is performed, where **** is $p<0.0001$.

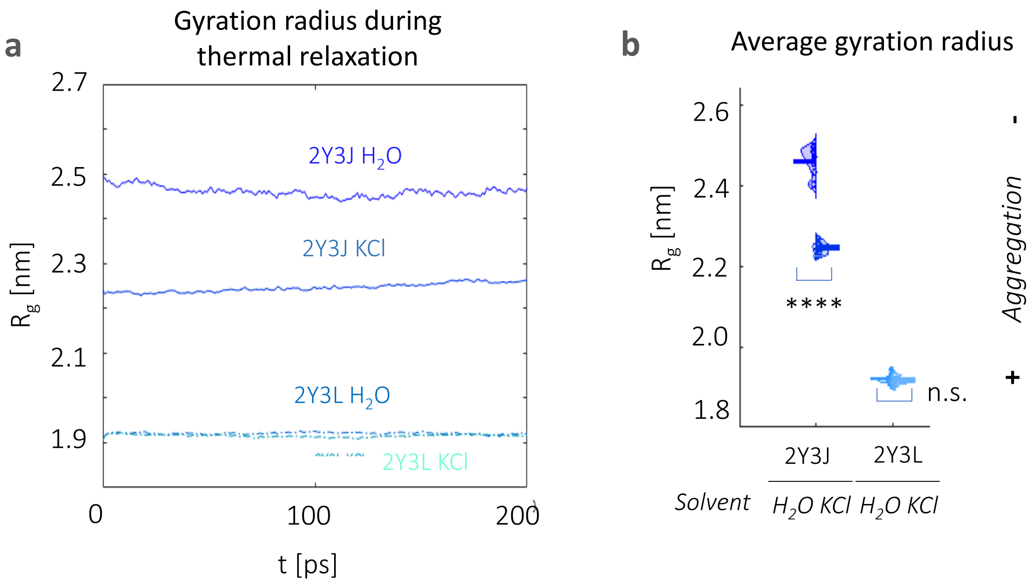

Supplementary Figure 7: 32C 2Y3J is more aggregated in KCl than in water. (a) Gyration radius traces for 32C 2Y3J and 2Y3L in water and in KCl during the thermal relaxation process. (b) 2Y3J takes on a significantly, whilst 2Y3L a slightly more compact structure in KCl compared to water. T-test, where n.s. is not significant and **** is $p<0.05$.

Supplementary table

Supplementary Table 1: Comparison of FPT-FLIM data where fluorescence lifetimes are quoted as mean of bi-exponential lifetimes (exponential fitting) and modulation lifetimes (phasor plot)

		Reported [7,8]	This work
Temperature range (°C)		24—42	24—45
Temperature resolution (°C)		0.5	0.8
Fluorescence lifetime [ns]	Exponential fitting*	5—8.5	5—7.5
	Phasor plots**	N/A	7.8—11.5

References

(1) Stephens, A. D.; Lu, M.; Fernandez-Villegas, A.; Kaminski Schierle, G. S. Fast Purification of Recombinant Monomeric Amyloid-β from E. Coli and Amyloid-β-m-Cherry Aggregates from Mammalian Cells. *ACS Chem. Neurosci* **2020**, *11*, 3213.

(2) Ovesný, M.; Křížek, P.; Borkovec, J.; Švindrych, Z.; Hagen, G. M. ThunderSTORM: A Comprehensive ImageJ Plug-in for PALM and STORM Data Analysis and Super-Resolution Imaging. *Bioinf.* **2014**, *30* (16), 2389–2390.

(3) Esbjörner, E. K.; Chan, F.; Rees, E.; Erdelyi, M.; Luheshi, L. M.; Bertoncini, C. W.; Kaminski, C. F.; Dobson, C. M.; Kaminski Schierle, G. S. Direct Observations of Amyloid β Self-Assembly in Live Cells Provide Insights into Differences in the Kinetics of Aβ(1-40) and Aβ(1-42) Aggregation. *Chem. Biol.* **2014**, *21* (6), 732–742.

(4) Paviolo, C.; Clayton, A. H. A.; Mcarthur, S. L.; Stoddart, P. R. Temperature Measurement in the Microscopic Regime: A Comparison between Fluorescence Lifetime- and Intensity-Based Methods. *J. Microsc.* **2013**, *250* (3), 179–188.

(5) Ranjit, S.; Malacrida, L.; Stakic, M.; Gratton, E. Determination of the Metabolic Index Using the Fluorescence Lifetime of Free and Bound Nicotinamide Adenine Dinucleotide Using the Phasor Approach. *J. Biophotonics* **2019**, *12* (11), e201900156.

(6) Ranjit, S.; Malacrida, L.; Jameson, D. M.; Gratton, E. Fit-Free Analysis of Fluorescence Lifetime Imaging Data Using the Phasor Approach. *Nat. Protoc.* **2018**, *13* (9), 1979–2004.

(7) Inada, N.; Fukuda, N.; Hayashi, T.; Uchiyama, S. Temperature Imaging Using a Cationic Linear Fluorescent Polymeric Thermometer and Fluorescence Lifetime Imaging Microscopy. *Nat. Protoc.* **2019**, *14*, 1293–1321.

(8) Hayashi, T.; Fukuda, N.; Uchiyama, S.; Inada, N. A Cell-Permeable Fluorescent Polymeric Thermometer for Intracellular Temperature Mapping in Mammalian Cell Lines. *PLoS ONE* **2015**, *10* (2), e0117677.

(9) Imamura, H.; Huynh Nhat, K. P.; Togawa, H.; Saito, K.; Iino, R.; Kato-Yamada, Y.; Nagai, T.; Noji, H. Visualization of ATP Levels inside Single Living Cells with Fluorescence Resonance Energy Transfer-Based Genetically Encoded Indicators. *Proc. Natl. Acad. Sci. USA* **2009**, *106* (37), 15651–15656.

(10) Jorgensen, W. L.; Tirado-Rives, J. The OPLS Potential Functions for Proteins. Energy Minimizations for Crystals of Cyclic Peptides and Crambin. *J. Am. Chem. Soc.* **1988**, *110* (6), 1657–1666.

(11) Smith, M. D.; Rao, J. S.; Segelken, E.; Cruz, L. Force-Field Induced Bias in the Structure of Aβ 21−30 : A Comparison of OPLS, AMBER, CHARMM, and GROMOS Force Fields. *J. Chem. Inf. Model* **2015**, *17*, 41.

(12) Nose, S. A Molecular Dynamics Method for Simulations in the Canonical Ensemble. *Mol. Phys.* **1983**, *52* (2), 255–268.

(13) Hoover, W. G. Canonical Dynamics: Equilibrium Phase-Space Distributions. *Phys. Rev. A* **1985**, *31* (3), 1695.

(14) Humphrey, W.; Dalke, A.; Schulten, K. VMD: Visual Molecular Dynamics. *J. Mol. Graph.* **1996**, *14* (1), 33–38.

(15) Kaminski Schierle, G. S.; van de Linde, S.; Erdelyi, M.; Esbjörner, E. K.; Klein, T.; Rees, E.; Bertoncini, C. W.; Dobson, C. M.; Sauer, M.; Kaminski, C. F. In Situ Measurements of the Formation and Morphology of Intracellular β-Amyloid Fibrils by Super-Resolution Fluorescence Imaging. *J. Am. Chem. Soc.* **2011**, *133* (33), 12902–12905.

(16) Lu, M.; Williamson, N.; Mishra, A.; Michel, C. H.; Kaminski, C. F.; Tunnacliffe, A.; Kaminski Schierle, G. S. Structural Progression of Amyloid-β Arctic Mutant Aggregation in Cells Revealed by Multiparametric Imaging. *J. Biol. Chem.* **2019**, *294* (5), 1478–1487. https://doi.org/10.1074/jbc.RA118.004511.

(17) National Center for Biotechnology Information. *Rhodamine B (Compound)*. National Center for Biotechnology Information (2022). PubChem Compound Summary for CID 6694, Rhodamine B. https://pubchem.ncbi.nlm.nih.gov/compound/Rhodamine-B. (accessed 2022-05-22).

(18) Kemnitz, K.; Yoshihara, K. Entropy-Driven Dimerisation of Xanthene Dyes in Non-Polar Solution and Temperature-Dependent Fluorescence Decay of Dimers. *J. Phys. Chem.* **1991**, *95* (16), 6095–6104.
